# Influence of Parotid Saliva Composition on Phosphate Species' Chemical State in Relation to Dental Caries

**DOI:** 10.1002/cre2.70089

**Published:** 2025-02-12

**Authors:** Angela Rovera, Ali S. Alzahrani, Guido Rovera, Paul Anderson

**Affiliations:** ^1^ Dental Physical Sciences Unit, Centre for Oral Bioengineering, Institute of Dentistry Queen Mary University of London London UK; ^2^ Department of Basic Medical, Oral and Allied Dental Sciences, College of Dentistry Taif University Taif Saudi Arabia; ^3^ Nuclear Medicine Division, Department of Medical Sciences University of Turin Turin Italy

**Keywords:** ^31^P‐NMR, dental caries, ICDAS, saliva composition

## Abstract

**Objectives:**

The aim of the present study was to analyze the influence of parotid saliva (PS) composition on the phosphate species' chemical state in relation to dental caries.

**Methods:**

Unilateral stimulated PS samples were collected from 21 healthy adult subjects using a Lashley cup. Clinical caries scores of each subject were classified using the ICDAS score. The pH was recorded for each saliva sample. The concentration of specific inorganic elements (K^+^, Na^+^, Ca^2+^, Al^3+^, Sr^2+^, Li^+^, Zn^2+^, and Mg^2+^) was measured using an Inductively Coupled Plasma‐Optical Emission Spectrometer (ICP‐OES). The PS effective concentration of ions and the degree of saturation (DS) with respect to enamel mineral were determined by speciation calculation. The PS chemical environment was determined using ^31^P‐Nuclear‐Magnetic‐Resonance‐Spectroscopy (^31^P‐NMR). Pearson's correlation test was applied to evaluate the influence of PS composition on the ICDAS score.

**Results:**

The pH varied from 5.9 to 7.6. The ICDAS scores varied from 0.03 to 2.48. PS was supersaturated with respect to HAp at all pH values. The ^31^P‐NMR peak position value reflects the phosphate chemical state within PS and its change in relation to pH. Only calcium ion concentration [Ca^2+^], aluminum activity {Al^3+^}, and DS_HAp_ significantly correlated with the ^31^P‐NMR peak position value (ppm), whereas no correlation was observed between the ^31^P‐NMR peak position value and the activities of K^+^, Na^+^, Sr^2+^, Mg^2+^, Li^+^, and Zn^2+^.

**Conclusions:**

This parotid saliva ^31^P‐NMR study has shown that calcium ion concentration [Ca^2+^], aluminum activity {Al^3+^}, and DS_HAp_ significantly influence the phosphate species' chemical state existing within PS and provides extended knowledge on the main biochemical determinants of the caries process.

## 1 Introduction

Saliva contains various phosphate species: phosphoric acid (H_3_PO_4_), as well as primary (H_2_PO_4_^−^), secondary (HPO_4_^−2^), and tertiary (PO_4_^−3^) inorganic phosphate ions (Tatevossian and Gould 1979). These phosphate species comprise about 80% of the total salivary inorganic phosphate, with about 10% to 25% complexed to inorganic ions, such as calcium, or bound to proteins (depending on the pH) (Grøn 1973). The proportion of each salivary phosphate species concentration is determined by the saliva pH (Edgar 1976).

The predominant forms of phosphate species within a physiological pH range are the hydrogen (HPO_4_^2^^−^) and dihydrogen (H_2_PO_4_^−^) phosphate (Fejerskov et al. 2015). An increase in saliva pH alters the proportion of the four phosphate species, such that there is a decrease in the level of the primary ion (H_2_PO_4_^−^), but an increase in the concentration of the tertiary ion (PO_4_^−3^), which is the most important ionic species with respect to the solubility of tooth mineral (Dawes and Weatherell 1990).

The solid phase of the dental enamel consists mainly of crystallized calcium ortho‐phosphates named hydroxyapatite (HAp) (Lagerlöf 1983). The enamel surface of a fully erupted tooth is in constant contact with saliva, which is saturated with respect to calcium phosphate salts, thereby maintaining the integrity of the enamel surface (Larsen and Pearce 2003). The degree of saturation with calcium phosphates is important in terms of the development of dental caries because of its fundamental role in maintaining the balance between hydroxyapatite remineralization and demineralization (McCann 1968). Furthermore, the rate and amount of enamel mineral dissolution depend not only on the pH but also on the concentration of calcium and phosphate ions in solution (Lagerlöf 1983; Hassanali et al. 2017a).

However, the enamel has a nonstochiometric apatite composition, with calcium substituted by a variety of metal cations (e.g., K^+^, Na^+^, Al^3+^, Sr^2+^, Mg^2+^, Li^+^, and Zn^2+^), with wide variations among elements due to the uniqueness of the in vivo microenvironment provided by saliva (Liu et al. 2013; Dawes 2003). This aspect is important as the ions' substitutions influence apatite solubility, particularly at low pH, and therefore caries susceptibility (Hassanali et al. 2017a).

Saliva pH decreases rapidly during an acidic challenge caused by bacterial metabolic products, or by ingestion of acidic foods. Below the critical pH of 5.5, the solubility isotherm of HAp shows that enamel and dentine mineral will demineralize (Dawes 2003). However, saliva contains calcium and phosphate ions, and acts as a natural buffer to neutralize acid and limit the dissolution process (Singh et al. 2015). As normal oral conditions return to above the critical pH of 5.5, and as the salivary concentrations of calcium and phosphate ions increase, the chemical reaction will be reversed, that is, calcium phosphates will be precipitated, and so demineralized tooth tissues are re‐mineralized (Anderson et al. 2001; Li et al. 2014).

The established critical pH is 5.5; the actual value depends on the concentrations and activities of OH^−^, Ca^2+^, and PO_4_^3^^−^ ions in the solution (Dawes 2003). The dissolution of bulk enamel is significantly influenced by its solubility product (Wang et al. 2006). Therefore, with sufficient supply of Ca^2+^, the enamel will not dissolve even at low pH, as long as the ion activity product (IAP) is equal to or greater than the solubility product constant of HAp (suggested to be pK_sp_ = 117.2) (Anderson et al. 2001). Solubility product values for the enamel have been reported to be in the range from 110 to 126 (Margolis and Moreno 1985; McDowell et al. 1977). This difference between values is due to the defective nature of the enamel lattice and the inclusion of impurities such as carbonate, magnesium, and sodium (Patel and Brown 1975).

Whole‐mouth saliva (WMS) is a complex fluid derived from the three major paired salivary glands and thousands of minor glands distributed throughout the oral cavity (Rovera, Hector, et al. 2022). It also contains bacteria as well as host‐derived cells. This cellular content of WMS can obscure the analysis because of the ongoing metabolic activity of oral bacteria and neutrophils (Gardner et al. 2019). However, this issue can be eliminated by the aseptic collection of saliva directly from the parotid salivary gland, where, if collected appropriately, the saliva is sterile (Rovera, Hector, et al. 2022).

The aim of this study was to evaluate the influence of parotid saliva composition on the phosphate species' chemical state in relation to dental caries.

## 2 Materials and Methods

### 2.1 Parotid Saliva (PS) Collection and Preparation

PS samples were collected by the same dentist from 21 subjects (11 males and 10 females), aged between 27 and 38 years. The exclusion criteria comprised smokers, pregnant women, current users of any regular medication or therapy, dry mouth symptoms, and acute illness within the 2 weeks preceding the start of the study. In addition, subjects were excluded from the study if they presented with clinically significant abnormalities in clinical chemistry or hematology, or if there was evidence of a risk of transmitting the agents responsible for acquired immune deficiency syndrome, and hepatitis B or C. Further, subjects who reported excessive intake of alcohol, defined as regular maximum weekly intake of greater than 28 units, were also excluded from the study (Sreebny and Schwartz 1986).

Subjects were asked not to eat or drink anything except water, follow oral hygiene practices, or undertake strenuous physical activity at least 2 h before saliva collection.

Unilateral chew‐stimulated PS sample collection was performed using a Lashley cup placed over the Stenson's duct (Lashley 1916). PS samples were collected from each subject into sterilized low‐affinity conical plastic collection tubes (Fisher Scientific) in 1.0 mL of paraffin oil to avoid CO_2_ loss (Bardow et al. 2000). The time of collection was fixed between 9 and 11 a.m. All saliva tubes were kept on ice from the moment of collection and immediately frozen (−35°C) for 3 days (Chiappin et al. 2007). Recent findings indicate that saliva components are resilient to freezing (Gardner et al. 2018). Thawed PS shows only a very small amount of precipitate (compared to submandibular and sublingual saliva), suggesting that this was not important as the focus of the study is not the protein component (Francis et al. 2000).

Subsequently, the PS samples were thawed and centrifuged immediately before pH and inductively coupled plasma optical emission spectroscopy (ICP‐OES) analyses; both analyses were carried out on the same day (Gardner et al. 2018).

### 2.2 International Caries Detection and Assessment System (ICDAS)

The severity of caries of each subject was classified according to the seven‐grade ICDAS scale (Pitts 2004). The mean caries score for each subject was calculated by dividing the total ICDAS scores by the number of all ICDAS scored teeth.

### 2.3 Inductively Coupled Plasma Optical Emission Spectroscopy (ICP‐OES)

Total ion concentrations in PS were detected and quantified using ICP‐OES (ICP; Varian Vista‐PRO. Varian Ltd, Oxford, UK). Each of the 21 PS samples was processed by acid digestion: 1 mL of PS, 2% HNO_3_, and 8.8 mL of pure deionized water. The ICP‐OES analysis was used to detect K^+^, Na^+^, Ca^2+^, Li^+^, Al^3+^, Mg^2+^, Sr^2+^, and Zn^2+^ in PS. The accuracy of the ICP‐OES analytical protocol was evaluated by the analysis of certified reference standard materials.

### 2.4 Speciation Calculation of PS Ion Activity

Despite the central importance of knowing the full speciation of saliva in order to predict its behavior, it is generally not possible to carry out a speciation analysis using analytical chemistry methods alone because salivary concentrations of most metals of interest are low and also because many relevant forms of metals cannot be measured directly.

Thus, in this study, chemical speciation determination was carried out using an analytical method (ICP‐OES) in conjunction with chemical speciation calculations.

PS ion activity (K^+^, Na^+^, Ca^2+^, Li^+^, Al^3+^, Mg^2+^, Sr^2+^, and Zn^2+^) was calculated using Equations ((1), (2), (3)):

(1) {a}=γ[c],

a, activity,

c, concentration, and

γ, activity coefficient.

The activity coefficients (*γ*) were calculated using Davies' modification of the extended Debye–Huckel Equation and the Debye factor (Carey and Vogel 2000).

(2) $$\log\gamma_{i}=-A{\times \;z}^{2}\times \left(\frac{\sqrt{I}}{1+\sqrt{I}}-0.3\times I\right),$$

*γ*, activity coefficient,

*I*, ionic strength, and

*z*, ion charge,

and

(3) $$I=\frac{1}{2}\sum \;c_{i}\times z_{i}^{2},$$

*c*, concentration of each ion (*i*).

### 2.5 Calculation of PS Phosphate Species Activity

The activity of phosphate pH‐dependent forms (phosphoric acid {H_3_PO_4_}, dihydrogen phosphate ions {H_2_PO_4_^−^}, hydrogen phosphate ions {HPO_4_^2^^−^}, and phosphate ions {PO_4_^3^^−^}) was calculated using Carey and Vogel (2000), simplified calculation (Carey and Vogel 2000).

(4) $$\{{\text{HPO}}_{4}^{2-}\}=\frac{{{[\text{PO}}_{4}]}_{\text{tot}}}{\frac{10^{-\text{pH}}}{{\text{Kp}}_{2}\times \gamma_{H_{2}{\text{PO}}_{4}^{-}}}+\frac{1}{\gamma_{H{\text{PO}}_{4}^{2-}}}},$$

where K_P2_ = 6.46 × 10^−8^, γ_H2PO4‐_ = 0.7589, γ_HPO42‐_ = 0.3318, and

(5) $$\{{\mathrm{PO}}_{4}^{3-}\}=\frac{{\mathrm{Kp}}_{3}\times \{{\mathrm{HPO}}_{4}^{2-}\}}{10^{-\text{pH}}},$$

where K_P3_ = 4.47 × 10^−13^.

### 2.6 Calculation of the Degree of Saturation of PS With Respect to Calcium Phosphate Salts

The degree of saturation of a solution with respect to calcium phosphate minerals was defined according to the equation of Anderson et al. (2001).

(6) $$\mathrm{DS}={\left(\frac{\mathrm{IAP}}{K_{\mathrm{sp}}}\right)}^{\frac{1}{n}},$$

where IAP = ion activity product, K_sp_ = solubility product constant of 5.52 × 10^−118^, corresponding to pKsp 117.2 (negative logarithm to the base 10 of the Ksp), as suggested by Hassanali et al. (Hassanali et al. 2017b, 2017a; Liu et al. 2013), and *n* = number of ions. If DS is greater than 1 (IAP > Ksp), the solution is supersaturated with respect to enamel mineral. If DS is less than 1 (IAP < Ksp), the solution is undersaturated. If DS = 1 (IAP = Ksp), the solution is just saturated with respect to HAp, and there will be no net dissolution or precipitation.

### 2.7 ^31^P‐NMR Spectroscopy Sample Preparation and Acquisition

Aliquots (600 μL) of each PS sample were filtered using a glass wool pipette and then mixed with deuterium oxide 10% (D_2_O) (Deuterium Oxide, D, 99.9% Cambridge Isotope Laboratories, USA) as a lock signal. Saliva ^31^P solution NMR spectra were acquired using a Bruker 400 MHz spectrometer (Bruker Biospin, Karlsruhe, Germany) in Fourier transform mode, with the sample temperature set at 26°C, water suppression using presaturation, four dummy scans to establish equilibrium, 64 scans collected and added together, width of 14 ppm, 90° pulse (8.25 µs), total delay of 5.9 s, relaxation delay *d*_1_ of 2 s, acquisition time of 3.9 s, number of points acquired 65,536 (64 K), and number of processed points 131,072 (128 K). The ^31^P‐NMR spectra were processed, and the chemical shift of each peak was assigned (in part per million, ppm) using Bruker TopSpin software (Bruker Biospin, Rheinstetten, Germany, version 4.0.5).

### 2.8 Statistical Analysis

Anonymized data regarding subjects were stored and queried using a relational database (Rovera, Fariselli, et al. 2022). Recorded data were analyzed using SPSS Statistics (IBM, USA). Distribution analysis was performed using the Shapiro–Wilk test, as it is a more appropriate method for small sample sizes (*N* = 21). The association between all the variables were analyzed using Pearson's correlation (2‐tailed) to calculate the statistical significance of each association when variables were normally distributed. The associations between variables were analyzed using Spearman's correlation coefficient to calculate the statistical association when variables were not normally distributed, skewed, and extreme values were present.

## 3 Results

**Figure 1:**
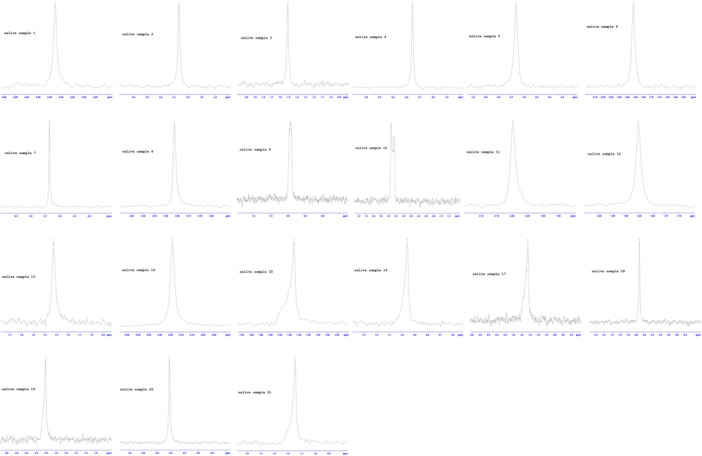
^31^P‐NMR spectrum of the 21 parotid saliva samples.

Figure 1 shows the variations in the phosphorous peak position between subjects' stimulated PS, suggesting variations in the phosphate states. It has been previously reported that different phosphate states exist within saliva and the ^31^P‐NMR chemical shift reflects the PS phosphate chemical state and its change between subjects (Larsen and Pearce 2003; Rovera et al. 2020).

Table 1 shows subjects' caries score and PS ion concentrations (mmol/l) ordered by ICDAS.

**Table 1:** Subjects' parotid saliva (PS) inorganic composition detected by ICP‐OES expressed in mmol/l, and caries susceptibility (ICDAS).

*n*	ICDAS	[K^+^]	[P_i_]	[Na^+^]	[Ca^2+^]	[Li^+^]	[Al^3+^]	[Mg^2+^]	[Sr^2+^]	[Zn^2+^]
1	0.03	10.22	4.49	3.8	0.66	0.01	0.01	0.03	0.00	0.00
2	0.07	12.73	6.76	1.33	0.67	0.01	0.01	0.03	0.00	000
3	0.07	13.88	6.56	11.01	1.62	1.15	0.32	0.03	0.04	0.02
4	0.1	12.54	6.47	7.77	1.86	1.12	0.31	0.11	0.04	0.02
5	0.11	6.28	6.73	3.7	0.95	1.1	0.33	0.04	0.05	0.03
6	0.25	10.66	6.52	4.05	1.22	1.09	0.31	0.04	0.04	0.02
7	0.35	11.79	6.56	3.77	1.37	1.09	0.31	0.04	0.05	0.02
8	0.39	4.72	6.9	5.54	1.07	1.13	0.34	0.04	0.05	0.02
9	0.43	2.92	1.22	0.09	0.25	0.01	0.01	0.01	0.00	0.00
10	0.46	13.51	6.58	1.85	2.14	0.01	0.01	0.03	0.00	0.01
11	0.53	18.86	10.58	0.73	1.69	0.01	0.02	0.11	0.00	0.00
12	0.62	12.63	6.56	5.28	2.18	1.12	0.31	0.04	0.04	0.02
13	0.64	5.3	1.39	0.28	0.28	0.01	0.01	0.02	0.00	0.00
14	0.64	13.37	6.54	1.08	1.29	0.01	0.07	0.05	0.00	0.01
15	0.67	11.65	5.97	0.76	0.83	0.01	0.01	0.06	0.00	0.00
16	0.72	15.01	4.72	4.19	1.29	0.01	0.01	0.02	0.00	0.00
17	1.14	12.31	6.81	1.73	1.39	0.01	0.01	0.05	0.00	0.01
18	1.18	16.73	8.72	7.53	1.36	0.01	0.03	0.12	0.00	0.01
19	1.36	8.92	2.94	2.52	0.92	0.01	0.01	0.1	0.00	0.00
20	1.8	11.22	6.64	3.4	1.57	1.12	0.32	0.06	0.05	0.02
21	2.48	14.11	7.71	0.7	1.05	0.01	0.02	0.08	0.00	0.01
Min	0.03	2.92	1.22	0.09	0.25	0.01	0.01	0.01	0.00	0.00
Max	2.48	18.86	10.58	11.01	2.18	1.15	0.34	0.12	0.05	0.03
Mean	0.66	11.4	6.07	3.39	1.22	0.43	0.13	0.05	0.02	0.01

*Note:* Data are ordered according to ICDAS.

Table 2 shows subjects' caries score and PS ion activity (mmol/l) ordered by ICDAS.

**Table 2:** Subjects' PS ion activity (mmol/l).

*n*	ICDAS	{Al}	{Mg}	{Sr}	{Li}	{Zn}	{Na}	{K}	{Ca^2+^}
1	0.03	3.20 × 10^−6^	2.63 × 10^−3^	2.29 × 10^−4^	4.46 × 10^−3^	1.85 × 10^−3^	3.13	8.39	5.40 × 10^−1^
2	0.07	5.60 × 10^−6^	1.44 × 10^−1^	2.04 × 10^−2^	9.42 × 10^−1^	1.13 × 10^−2^	9.05	1.140	2.40 × 10^−1^
3	0.07	6.00 × 10^−7^	3.41 × 10^−3^	2.27 × 10^−4^	4.62 × 10^−3^	1.41 × 10^−3^	1.09	1.050	4.90 × 10^−1^
4	0.10	3.50 × 10^−6^	1.40 × 10^−1^	2.00 × 10^−2^	9.21 × 10^−1^	1.10 × 10^−2^	6.39	1.030	5.30 × 10^−1^
5	0.11	2.00 × 10^−7^	1.50 × 10^−1^	2.22 × 10^−2^	9.01 × 10^−1^	1.32 × 10^−2^	3.04	5.16	3.20 × 10^−1^
6	0.25	4.50 × 10^−6^	1.40 × 10^−1^	2.05 × 10^−2^	8.98 × 10^−1^	1.03 × 10^−2^	3.33	8.76	2.70 × 10^−1^
7	0.35	6.00 × 10^−7^	1.41 × 10^−1^	2.06 × 10^−2^	8.99 × 10^−1^	9.59 × 10^−3^	3.10	9.69	3.90 × 10^−1^
8	0.39	1.00 × 10^−7^	1.53 × 10^−1^	2.30 × 10^−2^	9.32 × 10^−1^	1.13 × 10^−2^	4.55	3.88	5.70 × 10^−1^
9	0.43	3.00 × 10^−7^	3.21 × 10^−3^	2.59 × 10^−4^	4.71 × 10^−3^	2.20 × 10^−3^	7.14 × 10^−2^	2.40	8.20 × 10^−1^
10	0.46	4.20 × 10^−6^	3.96 × 10^−3^	3.07 × 10^−4^	4.52 × 10^−3^	3.87 × 10^−3^	1.50	1.110	4.60 × 10^−1^
11	0.53	2.00 × 10^−7^	6.86 × 10^−3^	1.74 × 10^−4^	4.45 × 10^−3^	1.52 × 10^−3^	6.00 × 10^−1^	1.550	3.50 × 10^−1^
12	0.62	2.90 × 10^−6^	1.43 × 10^−1^	2.04 × 10^−2^	9.19 × 10^−1^	9.80 × 10^−3^	4.34	1.040	5.40 × 10^−1^
13	0.64	4.30 × 10^−6^	3.20 × 10^−2^	1.97 × 10^−4^	4.33 × 10^−3^	6.34 × 10^−3^	8.86 × 10^−1^	1.100	6.60 × 10^−1^
14	0.64	0.00	2.76 × 10^−3^	2.52 × 10^−4^	4.70 × 10^−3^	1.85 × 10^−3^	2.32 × 10^−1^	4.350	7.30 × 10^−1^
15	0.67	9.00 × 10^−7^	3.01 × 10^−3^	2.45 × 10^−4^	4.75 × 10^−3^	2.22 × 10^−3^	6.25 × 10^−1^	9.570	4.60 × 10^−1^
16	0.72	3.60 × 10^−6^	5.79 × 10^−3^	7.10 × 10^−5^	4.18 × 10^−3^	1.04 × 10^−3^	3.44	1.230	7.30 × 10^−1^
17	1.14	1.40 × 10^−6^	4.06 × 10^−3^	3.40 × 10^−4^	4.83 × 10^−3^	3.70 × 10^−3^	1.43	1.010	3.40 × 10^−1^
18	1.18	0.00	1.57 × 10^−2^	1.32 × 10^−4^	4.13 × 10^−3^	3.78 × 10^−3^	6.19	1.370	5.80 × 10^−1^
19	1.36	4.00 × 10^−7^	3.67 × 10^−3^	1.44 × 10^−4^	4.48 × 10^−3^	3.22 × 10^−4^	2.07	7.330	1.60 × 10^−1^
20	1.80	1.00 × 10^−7^	1.46 × 10^−1^	2.12 × 10^−2^	9.17 × 10^−1^	1.08 × 10^−2^	2.79	9.220	4.40 × 10^−1^
21	2.48	0.00	7.14 × 10^−3^	1.07 × 10^−4^	4.44 × 10^−3^	2.46 × 10^−3^	5.75 × 10^−1^	1.160	1.40 × 10^−1^
Min	0.03	0.00	2.63 × 10^−3^	7.10 × 10^−5^	4.13 × 10^−3^	3.22 × 10^−4^	7.14 × 10^−2^	2.40	1.40 × 10^−1^
Max	2.48	5.60 × 10^−6^	1.53 × 10^−1^	2.30 × 10^−2^	9.42 × 10^−1^	1.32 × 10^−2^	9.05	1.550	8.20 × 10^−1^
Mean	0.6686	1.74 × 10^−6^	5.96 × 10^−2^	8.14 × 10^−3^	3.518 × 10^−1^	5.71 × 10^−3^	2.7827	9.3638	4.648 × 10^−1^

*Note:* Data are ordered according to the ICDAS score.

Table 3 shows the caries score for each subject and PS pH, ^31^P‐NMR chemical shift peaks (ppm), different phosphate moieties activity ({H_3_PO_4_}, {H_2_PO_4_^−^}, {HPO_4_^2^^−^}, {PO_4_^3^^−^}), calcium–phosphate complex activity ({CaHPO_4_°}, {CaH_2_PO_4_^+^}, {CaPO_4_^−^}), bicarbonate ion activity {HCO_3_^−^}, calcium hydrogen carbonate ion activity {CaHCO_3_^+^}, and calcium activity. The interindividual variations in PS ion activity varied markedly within the range from 5.85 to 7.60 found in this study.

**Table 3:** Subjects' caries susceptibility (ICDAS) and PS pH, ^31^P‐NMR chemical shift peaks (ppm), phosphate moiety activity ({H_3_PO_4_}, {H_2_PO_4_^−^}, {HPO_4_^2^^−^}, {PO_4_^3^^−^}), calcium–phosphate complex activity ({CaHPO_4_^0^}, {CaH_2_PO_4_^+^}, {CaPO_4_^−^}), bicarbonate ion activity {HCO_3_^−^}, calcium hydrogen carbonate ion activity {CaHCO_3_^+^}, calcium ion activity (mmol/l), and degree of saturation (DS) of parotid saliva (PS) with respect to Hydroxyapatite (HAp).

*n*	ICDAS	pH	^31^P‐NMR peak position value (ppm)	HCO^3^^−^	HPO_4_^2^^−^	PO_4_^3^^−^	H_2_PO_4_^−^	H_3_PO_4_	CaHCO_3_^+^	CaHPO_4_^0^	CaH_2_PO_4_^+^	CaPO_4_^−^	DS_HAp_
1	0.03	7.53	1.15	4.88	1.24	1.88 × 10^−^^5^	0.57	0.20 × 10^−^^5^	1.09 × 10^−^^4^	0.12	1.14 × 10^−^^3^	2.60 × 10^−^^2^	4.2
2	0.07	6.84	1.45	2.45	1.13	3.51 × 10^−^^6^	2.54	0.50 × 10^−^^4^	5.53 × 10^−^^5^	0.12	5.18 × 10^−^^3^	4.92 × 10^−^^3^	3.91
3	0.07	7.6	2.38	5.24	1.86	3.31 × 10^−^^5^	0.72	0.20 × 10^−^^5^	2.87 × 10^−^^4^	0.46	3.59 × 10^−^^3^	1.13 × 10^−^^1^	4.4
4	0.1	7.43	2.21	4.42	1.71	2.06 × 10^−^^5^	0.99	0.50 × 10^−^^5^	2.78 × 10^−^^4^	0.49	5.61 × 10^−^^3^	8.05 × 10^−^^2^	4.39
5	0.11	6.57	1.86	1.87	0.79	1.31 × 10^−^^6^	3.3	1.21 × 10^−^^4^	6.01 × 10^−^^5^	0.11	9.58 × 10^−^^3^	2.62 × 10^−^^3^	3.85
6	0.25	7.52	2.07	4.83	1.8	2.66 × 10^−^^5^	0.84	0.30 × 10^−^^5^	1.99 × 10^−^^4^	0.33	3.13 × 10^−^^3^	6.80 × 10^−^^2^	4.32
7	0.35	6.85	1.51	2.47	1.11	3.52 × 10^−^^6^	2.43	4.70 × 10^−^^5^	1.14 × 10^−^^4^	0.23	1.02 × 10^−^^2^	1.01 × 10^−^^2^	4.08
8	0.39	6.38	0.07	1.55	0.6	6.42 × 10^−^^7^	3.87	2.20 × 10^−^^4^	5.57 × 10^−^^5^	0.1	1.26 × 10^−^^2^	1.44 × 10^−^^3^	3.77
9	0.43	7.17	0.42	3.41	0.28	1.84 × 10^−^^6^	0.29	0.30 × 10^−^^5^	2.91 × 10^−^^5^	0.01	2.25 × 10^−^^4^	9.76 × 10^−^^4^	3.69
10	0.46	7.49	2.02	4.69	1.79	2.47 × 10^−^^5^	0.9	0.40 × 10^−^^5^	3.39 × 10^−^^4^	0.59	5.87 × 10^−^^3^	1.11 × 10^−^^1^	4.44
11	0.53	6.41	0.19	1.59	0.97	1.11 × 10^−^^6^	5.82	3.09 × 10^−^^4^	9.12 × 10^−^^5^	0.25	3.01 × 10^−^^2^	3.94 × 10^−^^3^	3.93
12	0.62	7.35	2.16	4.08	1.67	1.67 × 10^−^^5^	1.16	0.70 × 10^−^^5^	3.00 × 10^−^^4^	0.56	7.70 × 10^−^^3^	7.64 × 10^−^^2^	4.4
13	0.64	6.5	0.72	1.74	0.15	2.08 × 10^−^^7^	0.72	3.10 × 10^−^^5^	1.64 × 10^−^^5^	0.01	6.11 × 10^−^^4^	1.21 × 10^−^^4^	3.33
14	0.64	7.5	1.85	4.74	1.79	2.53 × 10^−^^5^	0.88	0.40 × 10^−^^5^	2.06 × 10^−^^4^	0.35	3.45 × 10^−^^3^	6.84 × 10^−^^2^	4.32
15	0.67	6.99	0.96	2.84	1.17	5.11 × 10^−^^6^	1.85	2.60 × 10^−^^5^	7.99 × 10^−^^5^	0.15	4.71 × 10^−^^3^	8.91 × 10^−^^3^	4.03
16	0.72	7.55	2.05	4.98	1.32	2.09 × 10^−^^5^	0.57	0.20 × 10^−^^5^	2.17 × 10^−^^4^	0.26	2.27 × 10^−^^3^	5.65 × 10^−^^2^	4.37
17	1.14	7.09	1.43	3.14	1.46	8.01 × 10^−^^6^	1.83	0.20 × 10^−^^4^	1.47 × 10^−^^4^	0.31	7.77 × 10^−^^3^	2.33 × 10^−^^2^	4.2
18	1.18	5.85	0.34	0.91	0.27	8.67 × 10^−^^8^	5.99	1.15 × 10^−^^3^	4.19 × 10^−^^5^	0.06	2.49 × 10^−^^2^	2.48 × 10^−^^4^	3.5
19	1.36	6.99	1.33	2.84	0.58	2.51 × 10^−^^6^	0.91	1.30 × 10^−^^5^	8.81 × 10^−^^5^	0.08	2.55 × 10^−^^3^	4.83 × 10^−^^3^	4
20	1.8	6.23	0.67	1.33	0.44	3.35 × 10^−^^7^	4.03	3.24 × 10^−^^4^	7.08 × 10^−^^5^	0.11	1.94 × 10^−^^2^	1.11 × 10^−^^3^	3.76
21	2.48	6.15	0.44	1.23	0.44	2.79 × 10^−^^7^	4.84	4.67 × 10^−^^4^	4.37 × 10^−^^5^	0.07	1.56 × 10^−^^2^	6.16 × 10^−^^4^	3.63
Min	0.03	5.85		0.91	0.15	8.67 × 10^−^^8^	0.29	2.00 × 10^−^^6^	1.64 × 10^−^^5^	0.01	2.25 × 10^−^^4^	1.21 × 10^−^^4^	3.33
Max	2.48	7.6		5.24	1.86	3.31 × 10^−^^5^	5.99	1.15 × 10^−^^3^	3.39 × 10^−^^4^	0.59	3.01 × 10^−^^2^	1.13 × 10^−^^1^	4.44
Mean	0.67	6.95		3.11	1.07	1.02 × 10^−^^5^	2.15	1.34 × 10^−^^4^	1.35 × 10^−^^4^	0.23	8.39 × 10^−^^3^	3.16 × 10^−^^2^	4.02

*Note:* Data are ordered according to the ICDAS score.

The activities of Ca^2+^, Al^3+^, HCO_3_^−^, CaHCO_3_^+^, HPO_4_^2^^−^, PO_4_^3^^−^, CaHPO_4_°, and CaPO_4_^−^ were all significantly positively correlated with the PS ^31^P‐NMR peak position value (ppm) (*p* < 0.05), whereas the activities of H_3_PO_4_, H_2_PO4^−^, and CaH_2_PO_4_^+^ were significantly negatively correlated with the ^31^P‐NMR peak position value (ppm) (*p* < 0.05).

**Figure 2:**
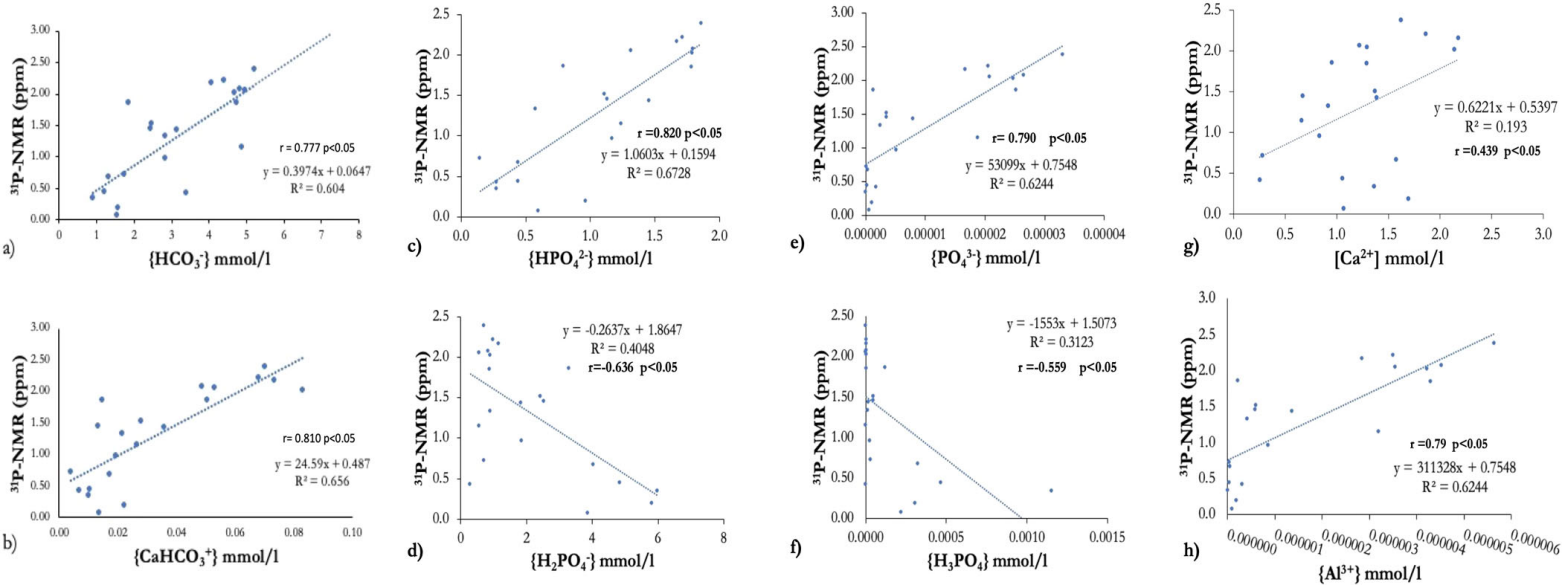
(a) Correlation of parotid saliva (PS) bicarbonate ion activity {HCO_3_^−^} (mmol/l) and (b) calcium hydrogen carbonate ion activity {CaHCO_3_} (mmol/l) with the ^31^P‐NMR peak position value (ppm); (c–f) correlation of PS phosphate ion activity (mmol/l) and ^31^P‐NMR peak position value (ppm); (g) correlation of PS calcium concentration [Ca^2+^] (mmol/l) and ^31^P‐NMR peak position value (ppm); and (h) correlation of PS aluminum activity {Al^3+^} (mmol/l) and ^31^P‐NMR peak position value (ppm).

The variations in the PS ^31^P‐NMR peak position value plotted as a function of (a) PS bicarbonate ion activity {HCO_3_^−^} and (b) calcium hydrogen carbonate ion activity {CaHCO_3_^+^} are shown in Figure 2. The phosphorus peak position value shifts toward positive values at high bicarbonate ion {HCO_3_^−^} and calcium hydrogen carbonate ion {CaHCO_3_^+^} activity (mmol/l).

Figure 2c–f shows variations in the ^31^P‐NMR peak position value plotted as a function of each PS phosphate species activity. The phosphorus peak shifts toward increased positive values with increasing hydrogen phosphate ion {HPO_4_^2^^−^} activity and phosphate ion {PO_4_^3^^−^} activity, but shifts toward negative values with increasing dihydrogen phosphate ion {H_2_PO_4_^−^} activity and phosphoric acid {H_3_PO_4_} activity.

Figure 2g shows the correlation between the PS ^31^P‐NMR peak position value (ppm) and the PS calcium concentration (mmol/l). The phosphorus peak position shifts toward positive values at high calcium concentrations.

Figure 2h shows the correlation between the PS ^31^P‐NMR peak position value (ppm) and PS aluminum activity (mmol/l). The phosphorus peak position shifts toward positive values at high aluminum activity.

**Figure 3:**
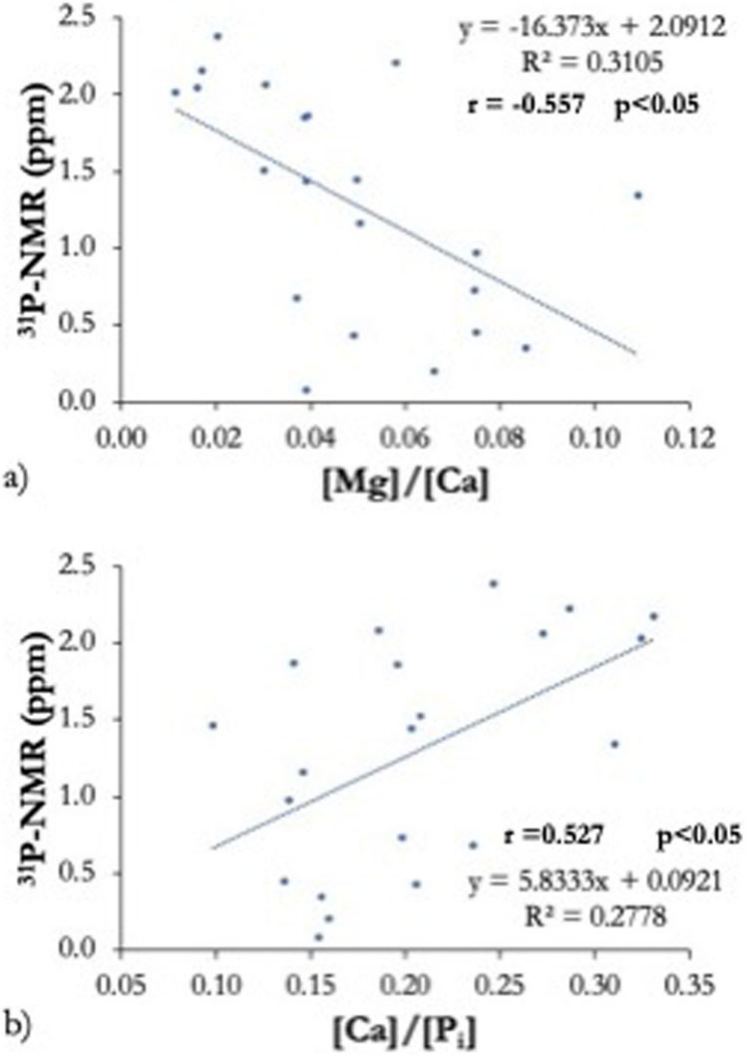
Correlation of parotid saliva (PS) ion ratios of [Mg]/[Ca], [Ca]/[P_i_], and the ^31^P‐NMR peak position value (ppm).

Significant Pearson's correlation coefficients were found between the PS ^31^P‐NMR peak position value (ppm) and ion ratios of [Mg]/[Ca] and [Ca]/[P_i_]. However, PS ^31^P‐NMR peak position values (ppm) were not significantly correlated with any of the other ratios, including [Sr]/[Ca], [Sr]/[Mg], [Zn]/[Ca], [Zn]/[Mg], and [Sr]/[Zn]. Figure 3 shows the variation in PS ^31^P‐NMR ppm plotted as a function of (a) the PS [Mg]/[Ca] ion ratio and (b) the [Ca]/[P_i_] ion ratio. The phosphorus peak position value (ppm) increases with decreasing [Mg]/[Ca] ratio and increasing [Ca]/[Pi] ratio.

**Figure 4:**
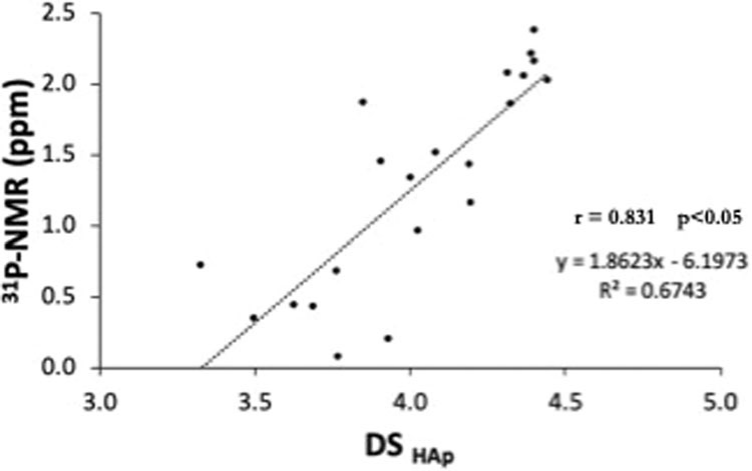
Correlation of parotid saliva (PS) saturation degree with respect to HAp (DS_HAp_) and the ^31^P‐NMR peak position value (ppm).

Figure 4 shows a plot of the PS ^31^P‐NMR peak position value (ppm) and the degree of saturation of PS with respect to enamel mineral.

Significant Pearson's correlation coefficients (*r*) and statistical significance (*p*) were found between PS ^31^P‐NMR ppm and the degree of saturation of PS with respect to HAp (DS_HAp_). The ^31^P‐NMR peak position value increases with DS_HAp_.

Table 4 shows that the PS ^31^P‐NMR peak position value correlated negatively with the ICDAS score.

**Table 4:** Pearson's correlation coefficients (*r*) and statistical significance (*p*) between PS ^31^P‐NMR peak position values (ppm) and the subjects' caries score (ICDAS).

	^31^P‐NMR	
	*r*	*p*
ICDAS	−0.43*	0.02

* Statistical significance at the 0.05 level.

## 4 Discussion

This ^31^P‐NMR study showed variations in the phosphorous peak position between subjects' stimulated PS, suggesting variations in the phosphate states. It has been previously reported that different phosphate states exist within saliva (Larsen and Pearce 2003; Rovera et al. 2020) and the ^31^P‐NMR chemical shift reflects the PS phosphate chemical state and its change between subjects.

### 4.1 Chemical Speciation

This study analyzed the influence of the inorganic chemical composition of PS on the phosphate speciation state, and then the consequential impact on caries susceptibility. The results demonstrate the importance of the degree of saturation of PS with respect to HAp on the specific phosphate species required to provide a protective and reparative dental hard tissue environment that is important for the integrity of the teeth.

The ^31^P‐NMR peak position value reflects the phosphate chemical state within PS and its change in relation to pH. The activities of HCO_3_^−^ and CaHCO_3_^+^ directly influence the salivary pH, which affects the phosphate chemical state, which in turn influences the caries process. If the salivary phosphate ion (PO_4_^3^^−^) and/or the hydroxyl ion (OH^−^) concentrations decrease sufficiently so that the ionic product falls below the solubility product for the tooth mineral, the tooth will demineralize in the acidified saliva (Dawes 2003).

This study also showed that the predominant phosphate species in stimulated PS within the normal physiological pH range are (1) dihydrogen phosphate (H_2_PO_4_^−^) and (2) hydrogen‐phosphate ion (HPO_4_^2^^−^), but not the phosphate ion (PO_4_^3^^−^). These two hydrogen phosphate species influence the ^31^P‐NMR peak position value (ppm) of PS. The HPO_4_^2^^−^ ion has been reported to be the most important phosphate species governing the solubility of hydroxyapatite because it is readily exchanged between saliva and the enamel surface (Chen and Wang 2010; Vogel et al. 1965). This is due to the very large specific surface areas of enamel crystals (resulting from their small dimension), thereby allowing the opportunity for its adsorption.

The ionized forms of calcium and phosphate in PS are important because both are constituents of the hydroxyapatite unit cell (Ca_10_(PO_4_)_6_(OH)_2_). The degree of saturation (DS) of PS with respect to calcium phosphate salts influences the solubility of HAp, thereby determining the rate at which the demineralization progresses. The ^31^P‐NMR peak position correlated significantly with the degree of saturation of PS with respect to HAp. The PS ^31^P‐NMR peak position shifts toward more positive values in subjects with a low caries score due to the high degree of saturation of their PS, which exerts an effect on both de‐ and remineralization processes.

Only calcium ion concentration [Ca^2+^] and aluminum activity {Al^3+^} significantly correlated with the ^31^P‐NMR ppm, whereas no correlation was observed between the ^31^P‐NMR peak position value and the activities of K^+^, Na^+^, Sr^2+^, Mg^2+^, Li^+^, and Zn^2+^.

This study showed that the ^31^P‐NMR peak position value become more positive with increasing PS calcium concentration [Ca^2+^] and aluminum activity {Al^3+^}. These results can be explained by the tendency of a hydrogen phosphate ion (HPO_4_^2^^−^) to selectively form complexes with a calcium ion (Ca^2+^) and the tendency of a phosphate ion (PO_4_^3^^−^) to form a complex with an aluminum ion (Al^3+^) at the physiological pH range found in PS.

Putt and Kleber (1995) showed that Al^3+^ is a cariostatic ion, and has an effect on both the oral microflora and the enamel mineral by increasing its acid resistance due to the formation of insoluble surface aluminum phosphate barriers and due to the substitution of two Al atoms for three calcium atoms in the hydroxyapatite lattice (Putt and Kleber 1995). The result of the present study is in agreement with the findings of Putt and Kleber (1995), as it is found that when there is higher PS aluminum {Al^3+^} activity, a more positive ^31^P‐NMR peak position value is obtained, and this is correlated with a lower caries score.

### 4.2 Ion Ratios

This study shows there is a correlation between the PS ^31^P‐NMR peak position value and the ion ratios of [Ca]/[P_i_] and [Mg]/[Ca]. Nucleation and growth of calcium phosphate are important aspects of remineralization and depend on the supersaturation and the ionic strength of saliva. Yoshihara et al. (2020) showed the influence of [Ca]/[P_i_] and [Mg]/[Ca] ion ratios on remineralization and on crystal formation in arrested caries lesions and reported the presence of Ca and P, as well as Mg within the HAp crystals deposited in caries‐arrested lesions (Yoshihara et al. 2020).

HAp formation is a complex process that can occur in a low Mg‐containing solution with a Mg/Ca ratio below 0.4 (Abbona and Franchini‐Angela 1990). The initial crystals formed during caries remineralization have been reported to be Mg‐containing ß‐tricalcium phosphate (Mg‐β‐TCP or whitlockite) and Mg‐containing HAp (Mg‐HAp) (Daculsi et al. 1979). In the present study, the Mg/Ca ratio of PS ranged between 0.01 and 0.11, so that the Mg^2+^ concentrations detected in PS might have facilitated HAP formation, thereby shifting the balance between de‐ and remineralization toward remineralization and favoring enamel preservation. These results are in agreement with those reported in the in vitro study of Yoshihara et al. (2020), who found a Mg/Ca ratio ranging from 0.01 to 0.08 in the intratubular HAp precipitates of dentinal carious lesions (Yoshihara et al. 2020).

## 5 Conclusion

This ^31^PNMR study has demonstrated the subtle role of the different phosphate chemical states within stimulated parotid saliva on enamel demineralization. These different phosphate chemical states significantly moderate saliva's inorganic chemical behavior in terms of the degree of chemical saturation with respect to enamel mineral, thereby influencing caries susceptibility, even above the so‐called “critical pH” value.

This ^31^PNMR study has also demonstrated the influence of PS calcium ion concentration [Ca^2+^] and aluminum ion activity {Al^3+^} on the phosphate chemical state and their role in the maintenance of the balance between de‐ and remineralization. Furthermore, the PS phosphate chemical state is also influenced by the [Ca]/[P_i_] and [Mg]/[Ca] ratios. When these ratios are low, the phosphate state shifts toward more favorable species (HPO_4_^2^^−^, PO_4_^3^^−^) for remineralization.

The results are based on a small sample and further research is warranted to further support the study findings.

## Author Contributions

Angela Rovera: Conceived the original idea. Carried out the experiments. Worked out all of the technical details and performed the numerical calculations for the experiment. Data collection and interpretation. Wrote the manuscript. Ali S. Alzahrani: Commented on the manuscript. Contributed to the writing of the manuscript. Designed the figures. Guido Rovera: Contributed to the design and implementation of the research, the analysis of the results, and writing of the manuscript. Analyzed spectra. Paul Anderson: Supervised the project. Contributed to the writing of the manuscript. Contributed to the design and implementation of the research.

## Ethics Statement

This study was carried out according to the ethical principles of Good Clinical Practice and the Declaration of Helsinki following approval from the Local Ethics Committee of ASO Santa Croce e Carle Cuneo, Italy (n.66‐17 of May 5, 2017). The subjects signed a written informed consent form before participating in the study.

## Conflicts of Interest

The authors certify that they have no affiliations with or involvement in any organization or entity with any financial interest (such as honoraria; educational grants; participation in speakers' bureaus; membership, employment, consultancies, stock ownership, or other equity interest; and expert testimony or patent‐licensing arrangements) or nonfinancial interest (such as personal or professional relationships, affiliations, knowledge, or beliefs) in the subject matter or materials discussed in this manuscript.

## Data Availability

Data are available in the article itself.
